# Artificial Intelligence for Preoperative Prediction of Lymph Node Metastasis and Depth of Invasion in Oral Tongue Squamous Cell Carcinoma: A Systematic Review and Meta-Analysis

**DOI:** 10.3390/diagnostics16050774

**Published:** 2026-03-04

**Authors:** Yi-Yun Ho, Chun-Wei Hsu, Ta-Yi Chu, Chun-Ju Lin, Yi-Hsin Ho, Cheng-Hsien Wu, Ching-Po Lin

**Affiliations:** 1 Oral & Maxillofacial Surgery, Department of Stomatology, Taipei Veterans General Hospital, Taipei 11217, Taiwan; puccaaccup1217@gmail.com (Y.-Y.H.); marcellinwu@gmail.com (C.-H.W.); 2 Faculty of Dentistry, Institute of Oral Biology, School of Dentistry, National Yang Ming Chiao Tung University, Taipei 112304, Taiwan; 3 Institute of Neuroscience, National Yang Ming Chiao Tung University, Taipei 112304, Taiwan; jht12020304@gmail.com; 4 Oral & Maxillofacial Surgery, Department of Stomatology, Chang Gung Memorial Hospital, Linkou 33305, Taiwan; bleedout972@gmail.com; 5 Department of Dentistry, School of Dentistry, National Yang Ming Chiao Tung University, Taipei 112304, Taiwan; grass5748@gmail.com; 6 Department of Dermatology, Taipei Veterans General Hospital, Taipei 11217, Taiwan; hoyh1217@gmail.com; 7 School of Medicine, National Yang Ming Chiao Tung University, Taipei 112304, Taiwan; 8 Center for Healthy Longevity and Aging Sciences, National Yang Ming Chiao Tung University, Taipei 112304, Taiwan; 9 Department of Education and Research, Taipei City Hospital, Taipei 10341, Taiwan

**Keywords:** artificial intelligence, radiomics, oral tongue squamous cell carcinoma, lymph node metastasis, depth of invasion

## Abstract

**Background**: Occult lymph node metastasis (OLNM) and depth of invasion (DOI) are key determinants of elective neck dissection in clinically node-negative oral tongue squamous cell carcinoma (OTSCC), yet accurate preoperative risk stratification remains challenging. This study evaluated the diagnostic performance of artificial intelligence (AI)-based predictive models for OLNM and DOI in OTSCC. **Methods**: A systematic review and meta-analysis were conducted in accordance with PRISMA 2020 guidelines. A structured search of PubMed identified twelve eligible studies, nine of which provided extractable 2 × 2 contingency data for inclusion in the primary bivariate meta-analysis. One additional study modeling DOI-derived pT stage was synthesized narratively. Pooled sensitivity and specificity were estimated using a bivariate random-effects model. Heterogeneity, threshold effects, and publication bias (Deeks’ test) were assessed. Methodological quality was evaluated using QUADAS-2 supplemented by an AI-specific methodological appraisal. **Results**: Across nine studies included in the primary meta-analysis, pooled sensitivity was 0.679 (95% CI: 0.604–0.745) and pooled specificity was 0.762 (95% CI: 0.705–0.811), with a summary AUC of 0.786. Heterogeneity was moderate for sensitivity (I^2^ = 41.8%) and low for specificity (I^2^ = 23.4%), with no significant threshold effect (ρ = −0.117, *p* = 0.776). No significant publication bias was detected (*p* = 0.596). Subgroup analyses showed comparable performance between OLNM-specific and general LNM models, whereas deep learning or hybrid approaches demonstrated higher accuracy than traditional machine learning methods. Notably, only one out of nine primary studies incorporated true external validation. **Conclusions**: AI-based models demonstrate moderate discriminative performance for predicting LNM and DOI in OTSCC and may serve as adjunctive tools in preoperative risk stratification rather than standalone decision-makers. However, the near absence of external validation, limited calibration reporting, and lack of clinician-comparator analyses substantially constrain current clinical translation. Future research should prioritize multi-center prospective validation, systematic calibration and decision-curve analyses, and adherence to TRIPOD-AI and CLAIM reporting standards.

## 1. Introduction

Oral tongue squamous cell carcinoma (OTSCC) represents a major clinical challenge and remains the most common and one of the most aggressive subtypes of oral cavity squamous cell carcinoma (OSCC). According to U.S. registry data, OTSCC accounts for approximately 41.7% of oral cavity SCC cases in the United States [1,2]. Its annual incidence has continued to increase, particularly among non-Hispanic White female individuals and younger patients, suggesting a shifting epidemiologic pattern [3]. In patients with clinically node-negative (cN0) necks, treatment planning remains complex. Although preoperative imaging and clinical examination may indicate node-negative status, a meaningful proportion of patients harbor occult lymph node metastasis (OLNM), which may lead to regional recurrence and reduced survival if unrecognized [4,5]. Conversely, performing elective neck dissection (END) in patients without pathologic nodal involvement may expose patients to unnecessary morbidity and long-term quality-of-life impairment, highlighting a persistent overtreatment-versus-undertreatment dilemma [4,5].

Depth of invasion (DOI) is another key prognostic factor and has been incorporated into the AJCC 8th edition T-classification for oral cavity SCC [6]. However, accurate preoperative estimation of DOI remains challenging in routine practice because of inherent imaging limitations and reliance on postoperative histopathologic assessment. To minimize conceptual ambiguity, this review distinguishes two related but clinically distinct endpoints. “OLNM” refers specifically to pathologically confirmed cervical nodal metastasis in patients who are clinically node-negative (cN0) on preoperative evaluation—the clinical scenario most directly relevant to elective neck dissection (END) decision-making. In contrast, “LNM” is used more broadly to describe cervical nodal metastasis in mixed cN0/cN+ cohorts, where the clinical objective concerns overall nodal staging rather than occult detection. This distinction is maintained consistently throughout the Methods, Results, and Discussion sections, and operational definitions for each included study.

Conventional imaging modalities, including computed tomography (CT) and magnetic resonance imaging (MRI), are widely used for nodal assessment and DOI evaluation in OTSCC; however, reported diagnostic performance has been inconsistent across studies [7,8,9]. With the increasing availability of high-dimensional imaging data and advances in computational capability, artificial intelligence (AI)—including machine learning (ML) and deep learning (DL)—has emerged as a potential adjunct in head and neck oncology [10,11,12,13]. By extracting quantitative image-derived features or learning hierarchical representations from imaging and clinical inputs, AI-based models have been developed for OLNM and DOI prediction.

Prior broader meta-analyses in oral and head-and-neck cancer settings reported pooled AUC values of approximately 0.89–0.92 for imaging-based LNM prediction; however, the direct applicability of these findings to OTSCC-specific cN0 and OLNM decision contexts remains uncertain [11,14]. Despite these encouraging findings, several barriers limit clinical translation. Many published models are derived from single-center cohorts with internal validation only, increasing the risk of overfitting and potentially inflating reported performance [10,15]. Furthermore, heterogeneity in imaging protocols, segmentation workflows, feature engineering strategies, model architecture, and evaluation metrics constrains cross-study comparability and robust synthesis. Although previous reviews have summarized AI applications in head and neck cancer, few have focused specifically on OTSCC, which has distinct biological behavior and treatment decision pathways [16].

Accordingly, an OTSCC-focused systematic review and meta-analysis of AI-based predictive models was conducted with prespecified endpoint separation. OLNM/LNM prediction was treated as the primary diagnostic endpoint for quantitative synthesis, whereas DOI-related prediction was summarized separately when direct pooling was methodologically inappropriate. Pooled diagnostic accuracy was estimated using a unified bivariate random-effects framework to derive summary sensitivity, specificity, and SROC AUC. Predefined subgroup analyses were performed to explore methodological factors associated with performance variability. In addition to QUADAS-2, an AI-specific methodological checklist was applied to provide complementary appraisal of clinical interpretability and translation readiness.

## 2. Materials and Methods

### 2.1. Search Strategy

A systematic literature search was conducted in PubMed (MEDLINE) in accordance with the Preferred Reporting Items for Systematic Reviews and Meta-Analyses (PRISMA 2020) guidelines. The completed PRISMA 2020 checklist is provided in the Supplementary Materials. The search encompassed all records from database inception through 15 January 2026.

The search strategy combined controlled vocabulary (Medical Subject Headings, MeSH) and free-text terms related to oral tongue squamous cell carcinoma (OTSCC), artificial intelligence, and diagnostic prediction.

Search terms encompassed disease-related keywords (“oral tongue squamous cell carcinoma”, “oral squamous cell carcinoma”, “OTSCC”, “OSCC”), imaging modalities (“magnetic resonance imaging”, “MRI”, “computed tomography”, “CT”, “PET/CT”), and analytical approaches (“artificial intelligence”, “machine learning”, “deep learning”, “radiomics”, “texture analysis”). Predictive outcome terms, including “depth of invasion”, “lymph node metastasis”, and “cervical lymph node”, were incorporated to ensure the comprehensive retrieval of relevant studies. Boolean operators (AND/OR) were applied to combine predefined concept blocks.

PubMed was selected as the primary database because it comprehensively indexes peer-reviewed biomedical literature in oncology, radiology, and medical informatics, which represent the core disciplines relevant to AI-based diagnostic modeling in OTSCC. Given the focused clinical scope of this review, PubMed provides a high coverage of relevant studies while maintaining specificity.

To further minimize retrieval bias, the reference lists of all included articles were manually screened for additional eligible studies.

No date or language restrictions were applied. The search was limited to human studies. The complete electronic search strategy is provided in Table A1.

### 2.2. Study Selection

Original studies were eligible if they applied machine learning (ML) or deep learning (DL) models to magnetic resonance imaging (MRI), computed tomography (CT), PET/CT imaging, or histopathological whole-slide imaging for the prediction of depth of invasion (DOI) or lymph node metastasis (LNM) in patients with OTSCC. PET/CT-based radiomic studies were explicitly included in both the search strategy and eligibility criteria; by Kudoh et al. [17] was identified and included. Only articles published in English were considered.

Eligible studies were required to provide a clear description of the imaging and analytical workflow and to report quantitative diagnostic performance metrics sufficient for evaluation. Histopathologic assessment served as the reference standard for DOI and/or nodal status when applicable.

Exclusion criteria comprised case reports, editorials, letters, reviews, meta-analyses, conference abstracts, and studies lacking sufficient methodological detail or outcome data. Articles not specifically focused on OTSCC or not employing ML/DL-based analytical frameworks were also excluded.

After the removal of duplicate records, two independent reviewers (Y.Y. HO and T.Y. CHU) screened titles and abstracts for relevance. Full-text articles were subsequently assessed for eligibility. Discrepancies were resolved through consensus discussion.

### 2.3. Data Extraction and Quality Assessment

Data were independently extracted from all eligible studies using a predefined data extraction form. Extracted variables included first author, year of publication, study design, imaging modality, sample size, and reference standard. Detailed characteristics of the artificial intelligence (AI) methodology were also collected, including model type, input configuration (imaging-only or multimodal), region-of-interest definition, preprocessing or feature extraction procedures, and validation strategy (internal versus external).

To address clinical heterogeneity in endpoint definitions, studies were classified into prespecified outcome subgroups according to harmonization rules. Studies were categorized as OLNM-specific when the study population was restricted to clinically N0 patients and the primary endpoint was histopathologically confirmed lymph node metastasis, either identified at elective neck dissection or during a defined follow-up period. Studies including mixed cN0/cN+ populations were classified as general LNM. When both overall and cN0-subgroup results were reported, the most clinically homogeneous dataset was selected for quantitative synthesis. DOI- or pT stage-based prediction models were analyzed separately in narrative form. These classification decisions were independently performed by two reviewers, with discrepancies resolved through consensus.

Diagnostic performance metrics—including sensitivity, specificity, true-positive, false-positive, true-negative, and false-negative counts, as well as areas under the receiver operating characteristic curve (AUC)—were extracted when available.

Methodological quality and risk of bias were independently assessed using the Quality Assessment of Diagnostic Accuracy Studies 2 (QUADAS-2) tool. Disagreements were resolved by consensus. In addition, an AI-specific methodological checklist (Table 1) was applied to evaluate eight domains critical for AI-based diagnostic models: segmentation reproducibility (ICC), class imbalance handling, external validation, calibration reporting, decision-curve analysis, clinician comparison, reporting guideline adherence, and code/data availability. A QUADAS-2 × AI cross-reference matrix (Table A2) was constructed to identify studies with low conventional risk of bias but important AI-specific methodological limitations.

Pooled sensitivity and specificity were jointly estimated using a bivariate random-effects model (Reitsma et al., 2005) [18,19], generating a summary receiver operating characteristic (SROC) curve. The threshold effect was assessed using Spearman rank correlation between logit(sensitivity) and logit(1—specificity). Between-study heterogeneity was described using I^2^ statistics for forest plots, while variance–covariance parameters from the bivariate model were considered the primary measure of between-study variability. Publication bias was evaluated using Deeks’ funnel plot asymmetry test [20].

Prespecified sensitivity analyses included: (a) leave-one-out analysis; (b) extended analysis incorporating methodologically borderline studies (k = 11); and (c) exclusion of mixed-site populations. The full search strategy is provided in Table A1. This review was conducted and reported in accordance with PRISMA 2020 and STARD principles and was registered in the PROSPERO database (CRD420251164051).

**Table 1:** AI-specific methodology checklist (N = 12 studies).

Study	AI-Specific Methodological Items								Score
	ICC Reported	Imbalance Handled	External Validation	Calibration	DCA	Clinician Comparison	Reporting Guideline	Code/Data Available	
Committeri et al. [21] ^†^	✗	✗	✗	✗	✗	✗	✗	✓ (Zenodo)	1/8
Csüry et al. [22] ^†^	✗	✗	✗	✗	✗	✗	✗	✗	0/8
Han et al. [23]	✗	✗	✗	✗	✗	✗	✗	✗	0/8
Kubo et al. [24]	✗	✓ (SMOTE)	✗	✗	✗	✗	✗	✗	1/8
Kudoh et al. [17]	✗	✗	✗	✗	✗	✗	✗	✗	0/8
Lan et al. [25]	✗	✓ (SMOTE)	✓ (True Ext)	✓	✓	✗	✓ (STARD)	✗	5/8
Liu et al. [26]	✓	✗	✗	✓	✗	✗	✗	✗	2/8
Vidiri et al. [27]	✓	✓ (SMOTE-NC)	✗	✗	✗	✗	✓ (TRIPOD)	✗	3/8
Wang et al. [28]	✗	✗	✗	✓	✓	✗	✗	✗	2/8
Wang et al. [29] ^†^	✓	✗	✗	✗	✗	✗	✗	✗	1/8
Yuan et al. [30]	✓	✗	✗	✗	✗	✗	✗	✗	1/8
Zhong et al. [31]	✓	✗	✗	✗	✗	✓ (NRI)	✗	✓ (GitHub)	3/8
**Total** **✓**	**5/12**	**3/12**	**1/12**	**3/12**	**2/12**	**1/12**	**2/12**	**2/12**	

**Definitions:** ICC, intraclass correlation coefficient (explicit reporting of a numeric reproducibility value required); Imbalance, explicit implementation of a class imbalance handling technique (e.g., SMOTE, class weighting, or resampling methods); External validation, evaluation on an independent external cohort from a different institution and/or acquired using different imaging equipment; Calibration, reporting of calibration assessment (e.g., calibration curve, Hosmer–Lemeshow test, or calibration slope); DCA, decision-curve analysis performed to evaluate clinical net benefit; Clinician comparison, formal quantitative head-to-head comparison between AI model performance and radiologist interpretation (e.g., NRI or equivalent metrics); Reporting guideline, explicit statement of adherence to TRIPOD, CLAIM, or STARD; Code/data availability, publicly accessible repository providing source code and/or dataset. Studies marked with ^†^ were excluded from the primary bivariate meta-analysis (*k* = 9). The total score represents the number of fulfilled AI-specific methodological items (maximum score = 8). Only Lan et al. [25] fulfilled the predefined criteria for true external validation.

## 3. Results

### 3.1. Literature Search and Study Selection

**Figure 1:**
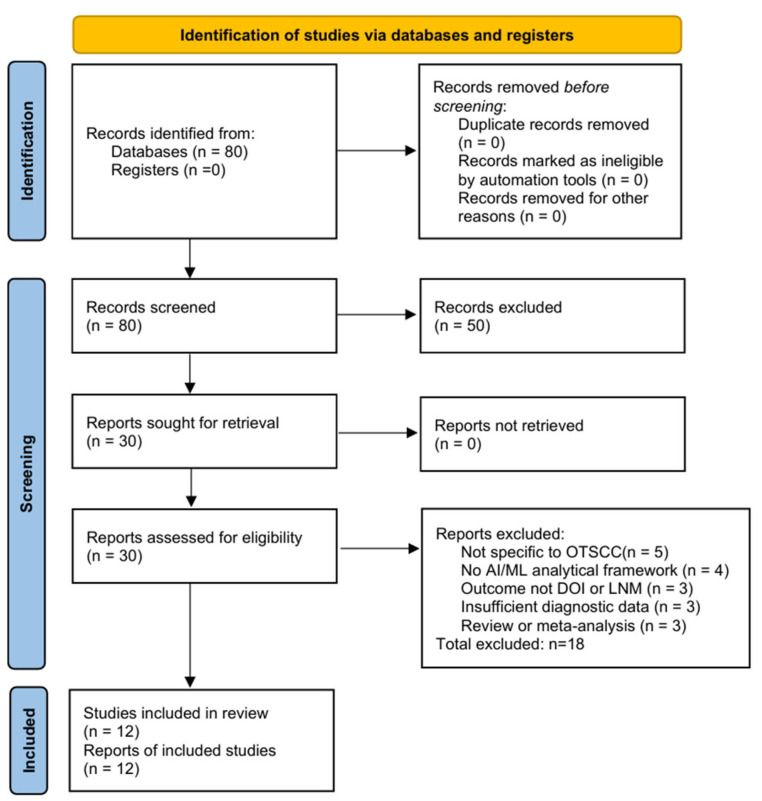
PRISMA flow diagram for study selection.

Figure 1 illustrates the PRISMA flow diagram of the literature search and study selection process. A total of 80 records were identified from PubMed. Following title and abstract screening, 50 records were excluded. Thirty full-text articles were assessed for eligibility, of which 18 were excluded for reasons detailed in Figure 1. Ultimately, twelve studies met the inclusion criteria and were included in the qualitative synthesis.

Of these, nine studies provided extractable 2 × 2 contingency data and were included in the primary bivariate meta-analysis. Three studies (Committeri et al. [21], Csüry et al. [22], Wang et al. [29]) were included in the systematic review but excluded from the primary quantitative synthesis due to high risk of bias, non-imaging input modality, or insufficient extractable data, respectively.

The imaging modalities represented were MRI (*n* = 5), CT (*n* = 3), 18F-FDG PET/CT (*n* = 1), and histopathological whole-slide imaging (*n* = 1). Sample sizes ranged from 25 to 319 patients. AI approaches comprised traditional machine learning models (*n* = 7), including support vector machines (SVM), random forests (RF), artificial neural networks (ANN), logistic regression, classification and regression decision trees (CIDT), and threshold-based methods, as well as deep learning or hybrid deep learning–radiomics models (*n* = 5), including LightGBM, multilayer perceptron (MLP), and ResNet50 combined with radiomics.

Operationalized endpoint definitions and subgroup classifications for all twelve studies are summarized in Table 2.

### 3.2. Quality Assessment

**Figure 2:**
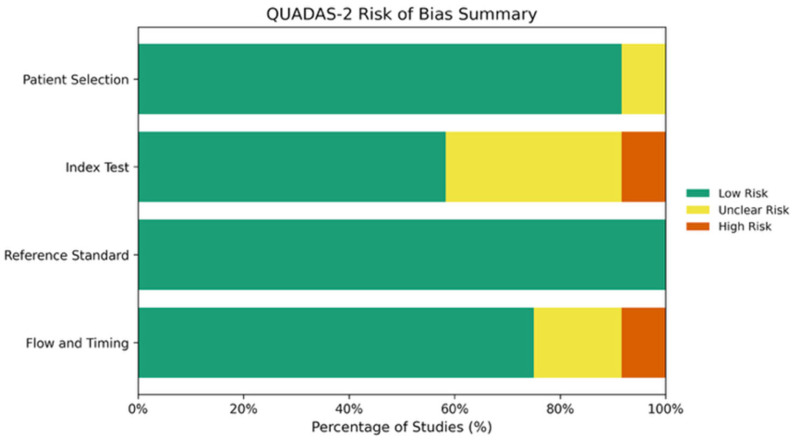
Quality assessment of included studies using the QUADAS-2 tool.

Figure 2 and Table 3 summarize the QUADAS-2 assessment of risk of bias and applicability across four domains: patient selection, index test, reference standard, and flow and timing.

Risk of bias for patient selection was rated as low in 11 out of 12 studies (91.7%), with one study judged unclear due to insufficient reporting of consecutive enrollment. All twelve studies (100%) were considered to have used an appropriate reference standard. The index test domain demonstrated the greatest methodological concern, with 7 out of 12 studies (58.3%) rated as low risk, 4 as unclear, and 1 as high risk (Committeri et al. [21]), primarily due to the absence of an independent validation set. Flow and timing was rated as low risk in 9 out of 12 studies (75.0%), unclear in 2, and high risk in 1 (Committeri et al. [21]).

Overall, applicability concerns were judged to be low across all studies, indicating that the included evidence was generally relevant to the review objectives.

Risk of bias and applicability concerns were evaluated across four domains: patient selection, index test, reference standard, and flow and timing. Overall, the majority of studies demonstrated a low risk of bias and low applicability concerns. Patient selection was rated low risk in 11/12 studies (91.7%), with one unclear. All twelve studies (100%) used an adequate reference standard. The index test domain showed the most variation: 7/12 low risk, 4/12 unclear, and 1/12 high risk (Committeri et al. [21]). Flow and timing was low risk in 9/12, with 2/12 unclear and 1/12 high risk. The summary bar chart indicates that 91.7% of studies were rated low risk for patient selection, 100% for reference standard, 58.3% for the index test, and 75.0% for flow and timing. All studies had low applicability concerns across all domains.

### 3.3. Methodological Quality Items

The methodological evaluation integrated QUADAS-2 with an AI-specific checklist to assess modeling transparency, validation strategy, and potential clinical utility. Overall, methodological rigor was moderate.

All twelve studies (100%) used postoperative histopathology as the reference standard, resulting in low risk of bias in that domain. Patient selection was rated as low risk in eleven studies (91.7%), with one study judged unclear due to insufficient reporting of consecutive enrollment. Potential bias was most frequently observed in the patient selection and index test domains, largely attributable to retrospective single-center designs and limited reporting of blinding between AI outputs and reference standard pathology.

Regarding model development, eleven studies (91.7%) constructed predictive models using radiomic or deep learning features derived from MRI, CT, or PET/CT. However, only five studies (41.7%; Yuan et al. [30], Zhong et al. [31], Zhong et al. [31], Vidiri et al. [27], Wang et al. [29]) assessed feature reproducibility or inter-reader reliability, and three explicitly addressed class imbalance. Eleven studies (91.7%) implemented data-driven feature selection strategies, including LASSO regression (*n* = 6), principal component analysis (*n* = 2), and statistical ranking methods (*n* = 3), whereas only one study (Csüry et al. [22]) applied end-to-end classification without explicit feature selection.

Internal validation was performed in eleven studies (91.7%), whereas only one study (8.3%) employed true external validation. Confidence intervals for sensitivity and specificity were reported in seven studies, and only one study used bootstrap or permutation resampling to estimate uncertainty.

Most studies did not report calibration analysis, decision-curve analysis, or head-to-head comparisons with radiologist interpretation, thereby limiting the assessment of clinical utility.

Cross-referencing QUADAS-2 with the AI-specific checklist (Table A2) revealed notable discordance. Six out of the nine primary meta-analysis studies (Yuan et al. [30], Zhong et al. [31], Han et al. [23], Liu et al. [26], Wang et al. [28], Lan et al. [25]) were rated as low risk across all four QUADAS-2 domains yet concurrently exhibited multiple AI-specific methodological gaps (ranging from 3 to 8 unfulfilled items out of 8). This pattern suggests that QUADAS-2 alone may be insufficient for a comprehensive evaluation of AI-based diagnostic models [32].

Among the twelve included studies, only Vidiri et al. [27] modeled DOI/pT stage as an AI-predicted outcome in addition to a separate LNM model. The DOI/pT model utilized MRI-derived radiomic features to classify tumors as pT1 versus pT2–pT3, achieving an AUC of 0.71 with a sensitivity of 0.667 and specificity of 0.714 in the test set (*n* = 36). Given that this represents a single study, quantitative synthesis was not performed. These findings are reported for completeness and should be interpreted cautiously in light of the limited sample size and absence of external validation.

### 3.4. Diagnostic Accuracy

**Figure 3:**
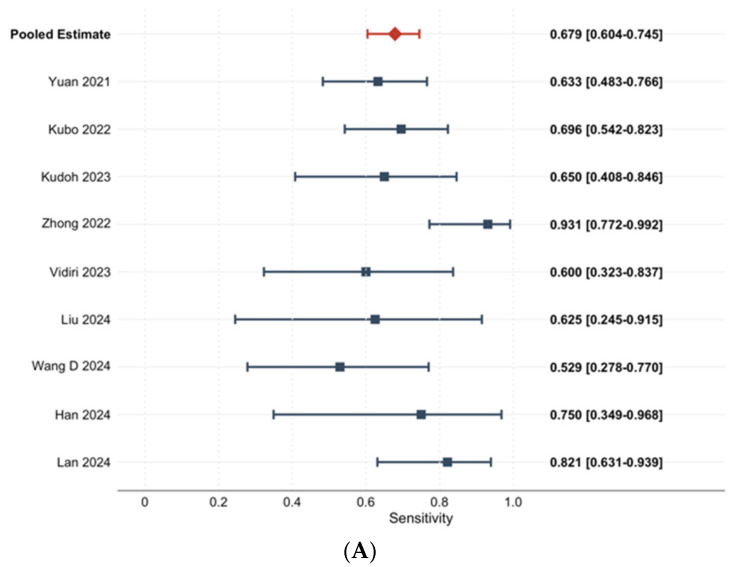

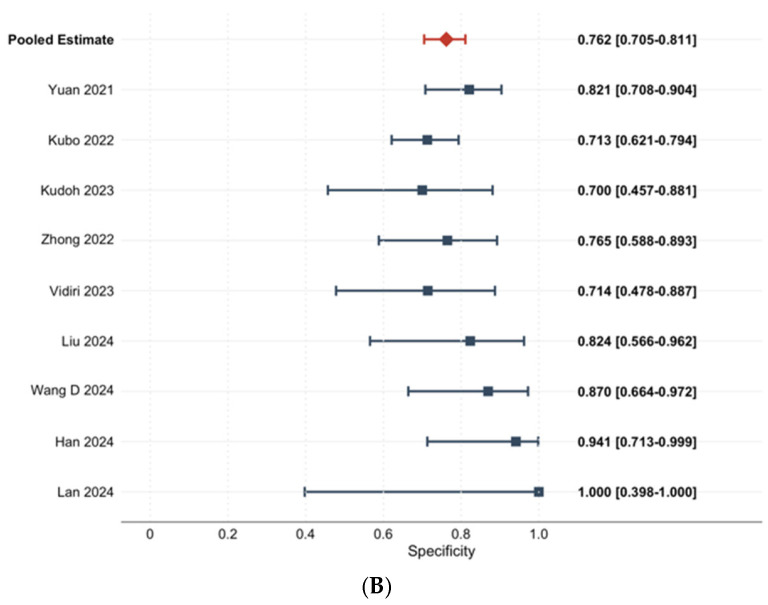
(**A**). Forest plot of sensitivity across included studies [17,23,24,25,26,27,28,29,30,31]. (**B**) Forest plot of specificity across included studies [17,23,24,25,26,27,28,29,30,31].

The diagnostic reference standards, endpoint definitions, and key performance metrics are summarized in Table 4. Nine studies providing extractable 2 × 2 contingency data were included in the primary bivariate meta-analysis. Individual and pooled sensitivity and specificity estimates are presented in Figure 3.

**Figure 4:**
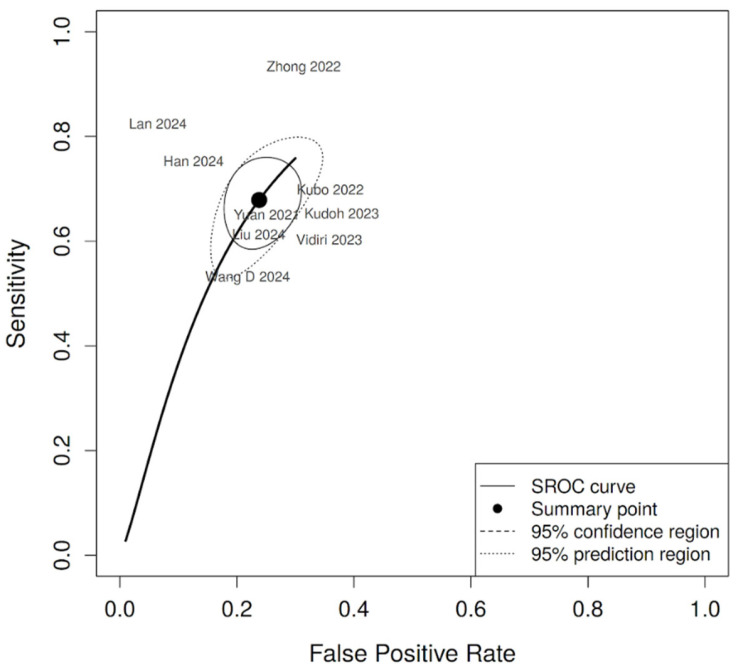
Summary receiver operating characteristic (SROC) curve of AI-based diagnostic models. The SROC curve was derived from the bivariate random-effects model (*k* = 9). Studies are labeled by first author and publication year. Each point represents an individual study (Yuan et al. [30], Kubo et al. [24], Kudoh et al. [17], Zhong et al. [31], Vidiri et al. [27], Liu et al. [26], Wang et al. [28], Han et al. [23], and Lan et al. [25]), and the solid curve represents the estimated summary ROC function. The summary area under the curve (AUC) was 0.786, with a summary operating point corresponding to a sensitivity of 0.679 and a specificity of 0.762.

The pooled sensitivity was 0.679 (95% CI: 0.604–0.745), and the pooled specificity was 0.762 (95% CI: 0.705–0.811). Between-study heterogeneity was moderate for sensitivity (I^2^ = 41.8%) and low for specificity (I^2^ = 23.4%). No significant threshold effect was observed (Spearman ρ = −0.117, *p* = 0.776). The summary ROC curve (Figure 4) yielded an AUC of 0.786, with a diagnostic odds ratio of 6.76, a positive likelihood ratio of 2.85, and a negative likelihood ratio of 0.42.

**Figure 5:**
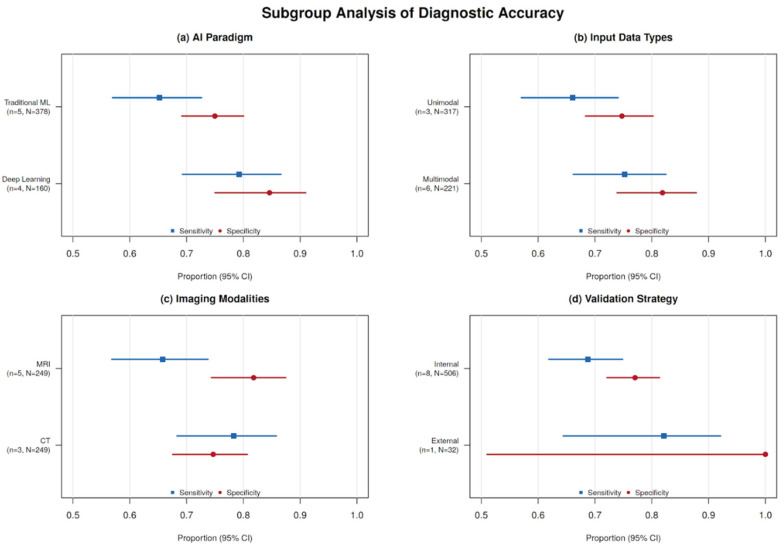
Subgroup analysis of diagnostic accuracy of AI-based models for predicting occult lymph node metastasis (OLNM) or depth of invasion (DOI) in oral tongue squamous cell carcinoma (OTSCC). Subgroup analyses were performed to explore potential sources of heterogeneity in diagnostic performance. Blue markers indicate sensitivity, and red markers indicate specificity, with horizontal lines representing the corresponding 95% confidence intervals (CI).

**Figure 6:**
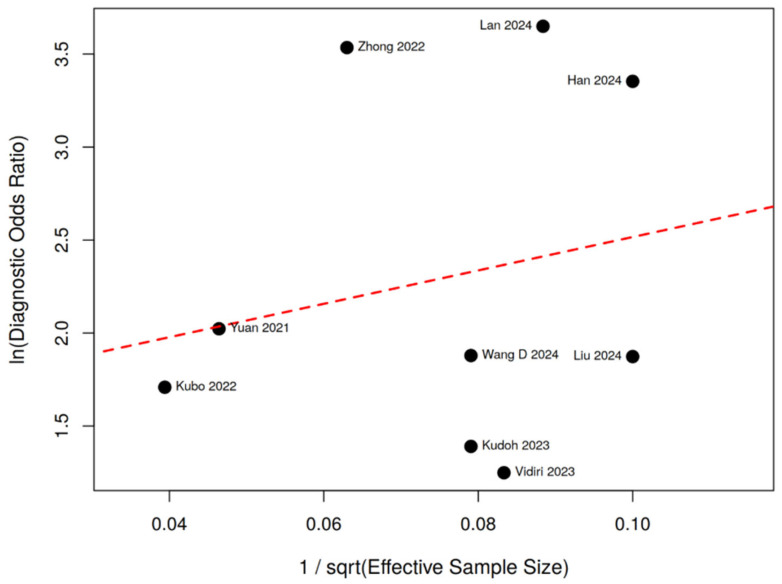
Deeks’ funnel plot for publication bias assessment in studies evaluating AI-based models for predicting lymph node metastasis in oral tongue squamous cell carcinoma (OTSCC). Studies are labeled by first author and publication year. Each point represents an individual study (Yuan et al. [30], Kubo et al. [24], Kudoh et al. [17], Zhong et al. [31], Vidiri et al. [27], Liu et al. [26], Wang et al. [28], Han et al. [23], and Lan et al. [25]). The dashed red line represents the regression line used in Deeks’ asymmetry test. No significant evidence of publication bias was detected (*p* = 0.596).

Subgroup analyses across AI paradigms, input types, imaging modalities, and validation strategies are summarized in Figure 5. The Deeks’ funnel plot (Figure 6) demonstrated no significant asymmetry (*p* = 0.596), although the statistical power of this test was limited with k = 9.

Exploratory subgroup analyses by outcome definition showed the following results: OLNM (k = 4), sensitivity = 0.690 (95% CI: 0.575–0.784) and specificity = 0.785 (95% CI: 0.668–0.868); general LNM (k = 5), sensitivity = 0.678 (95% CI: 0.571–0.769) and specificity = 0.769 (95% CI: 0.696–0.829). Deep learning or hybrid models (k = 4) achieved a sensitivity = 0.765 and specificity = 0.832, compared with traditional machine learning models (k = 5; sensitivity = 0.652, specificity = 0.750). MRI-based models (k = 5) showed a sensitivity = 0.643 and specificity = 0.802, whereas CT-based models (k = 3) showed a sensitivity = 0.806 and specificity = 0.749. Multimodal input studies (k = 6) yielded a sensitivity = 0.714 (95% CI: 0.569–0.826) and specificity = 0.815 (95% CI: 0.744–0.871) compared with unimodal approaches (k = 3; sensitivity = 0.666 [95% CI: 0.558–0.760], specificity = 0.712 [95% CI: 0.635–0.778]). Given the limited number of studies per subgroup (k = 3–6), formal statistical comparisons were not performed, and these findings should be interpreted as exploratory.

Only one of the nine primary studies (Lan et al. [25]) employed true external validation; therefore, a formal comparison between internal and external validation strategies was not undertaken. Leave-one-out sensitivity analysis demonstrated stable pooled estimates across all nine iterations (sensitivity range: 0.657–0.697; specificity range: 0.743–0.787), indicating no disproportionate influence of any single study.

An extended analysis incorporating methodologically borderline studies (Committeri et al. [21] and Csüry et al. [22]; k = 11) yielded a sensitivity = 0.688, specificity = 0.809, and AUC = 0.828. The modest increase in AUC was partly attributable to the high-performing Csüry et al. [22] model (histopathologic input, AUC = 0.83), indicating that inclusion of non-imaging models would inflate the apparent performance of imaging-based approaches. Exclusion of Kubo et al. [24] (delayed-detection OLNM definition) resulted in stable OLNM subgroup estimates (sensitivity = 0.685, specificity = 0.787). Similarly, exclusion of Lan et al. [25] (which included oropharyngeal SCC cases) yielded a sensitivity = 0.664, specificity = 0.770, and AUC = 0.766.

Overall, AI-based diagnostic models demonstrated moderate discriminative performance for the preoperative prediction of LNM in OTSCC. However, the limited use of external validation and substantial methodological heterogeneity indicate restricted readiness for clinical translation.

Forest plots showing the individual and pooled diagnostic performance of AI-based models for predicting lymph node metastasis (OLNM or general LNM) in oral tongue squamous cell carcinoma (OTSCC). Studies are labeled by first author and publication year (Yuan et al. [30], Kubo et al. [24], Kudoh et al. [17], Zhong et al. [31], Vidiri et al. [27], Liu et al. [26], Wang et al. [28], Han et al. [23], and Lan et al. [25]).

In the sensitivity analysis, each horizontal line represents the 95% confidence interval (CI) for an individual study, and the red diamond indicates the pooled estimate derived from the bivariate random-effects model. The pooled sensitivity was 0.679 (95% CI: 0.604–0.745; k = 9), with moderate between-study heterogeneity (I^2^ = 41.8%).

In the specificity analysis, squares represent point estimates for individual studies, with horizontal lines indicating the corresponding 95% CIs. The pooled specificity was 0.762 (95% CI: 0.705–0.811; k = 9), with low between-study heterogeneity (*I*^2^ = 23.4%).

Blue markers indicate sensitivity, and red markers indicate specificity, with horizontal lines representing the corresponding 95% confidence intervals (CI).

(a) AI paradigm: Deep learning or hybrid models (*k* = 4) demonstrated higher pooled sensitivity (0.765) and specificity (0.832) compared with traditional machine learning approaches (*k* = 5; sensitivity = 0.652, specificity = 0.750).

(b) Input data types: Multimodal input strategies (*k* = 6) yielded a pooled sensitivity of 0.714 and specificity of 0.815, whereas unimodal approaches (*k* = 3) showed a sensitivity of 0.666 and specificity of 0.712.

(c) Imaging modalities: MRI-based models (*k* = 5) achieved a pooled sensitivity of 0.643 and specificity of 0.802, while CT-based models (*k* = 3) demonstrated a higher pooled sensitivity (0.806) but slightly lower specificity (0.749).

(d) Validation strategy: Only one study (Lan et al. [25]) employed true external validation. Because of the limited number of studies within each subgroup (*k* = 3–6), formal statistical comparisons between subgroups were not performed. These subgroup analyses should therefore be interpreted as exploratory.

## 4. Discussion

This systematic review and bivariate meta-analysis demonstrate that artificial intelligence (AI)-based models show moderate potential for supporting preoperative risk stratification in oral tongue squamous cell carcinoma (OTSCC). The pooled sensitivity (0.679) and specificity (0.762) indicated a fair diagnostic performance, with a summary ROC AUC of 0.786. Compared with our initial analysis, the current estimates were slightly lower, reflecting the application of a more rigorous bivariate framework and the inclusion of studies reporting more conservative performance metrics [33]. These findings suggest that AI may serve as a complementary adjunct to conventional imaging and clinical assessment rather than a standalone decision-making tool.

Subgroup analyses demonstrated a consistent performance advantage of deep learning (DL) or hybrid architectures over traditional radiomics-based machine learning (ML) approaches [14]. From a methodological standpoint, DL models benefit from hierarchical feature learning and the ability to capture complex nonlinear spatial relationships directly from the imaging data, thereby reducing dependence on handcrafted radiomic descriptors. This architectural flexibility likely contributes to the superior pooled sensitivity and specificity observed in our analysis. However, this apparent advantage warrants cautious interpretation. Several included DL studies exhibited substantial train–test performance gaps—for example, Wang et al. [28] reported a ΔAUC of 0.232 between training and test cohorts—raising concerns regarding overfitting in small-sample medical imaging datasets. As emphasized by Varoquaux and Cheplygina [15], internal validation frameworks frequently produce optimistically biased performance estimates. Thus, the observed DL superiority may reflect both genuine modeling capacity and residual methodological inflation.

From a clinical perspective, multimodal models integrating imaging-derived features with clinical variables demonstrated improved diagnostic performance, particularly in specificity [10]. This finding aligns with real-world oral oncology practice, where decisions regarding elective neck dissection rely on integrated radiologic and clinical assessment. Studies such as Liu et al. [26] and Wang et al. [28] illustrate the incremental value of incorporating contrast-enhanced MRI sequences or clinical lymph node status into predictive pipelines. These observations are consistent with broader trends toward multi-input fusion architectures in medical imaging AI [34,35]. Similarly, MRI-based models tended to demonstrate higher specificity than CT-based models, consistent with MRI’s established advantage in soft-tissue delineation and depth of invasion (DOI) assessment in oral tongue lesions [36]. Nevertheless, given the limited number of studies per subgroup and absence of formal interaction testing, these findings should be interpreted as exploratory rather than definitive evidence of superiority.

Several methodological gaps identified in our structured quality assessment have direct clinical implications. Only 3 out of 12 studies reported calibration metrics, and none formally evaluated model interpretability using approaches such as SHAP or Grad-CAM [37,38]. Without calibration assessment, predicted probabilities cannot be reliably translated into clinically actionable decision thresholds—an essential requirement when considering elective neck dissection in cN0 patients. Likewise, the absence of interpretability analyses limits clinician trust and transparency. Only one study (Zhong et al. [31]) directly compared model performance against radiologist interpretation, reporting net reclassification improvement up to 40%. In the absence of head-to-head comparisons, the incremental clinical value of AI beyond expert judgment remains uncertain.

The near absence of true external validation represents a major limitation of the current evidence base [39]. After systematic re-verification, only one study (Lan et al. [25]) employed genuine external validation across independent hospital cohorts, whereas most studies relied on internal split-sample or cross-validation designs. Such validation strategies are known to inflate performance metrics in medical imaging AI. If similar optimism bias applies across studies, the true diagnostic performance of AI models for OLNM or DOI prediction may be lower than the pooled AUC of 0.786 observed in this analysis. Strengthening generalizability through multicenter prospective validation remains an urgent priority.

Adherence to reporting guidelines was limited. Only one study referenced TRIPOD, one referenced STARD, and none explicitly adhered to the CLAIM checklist for AI in medical imaging [40,41,42]. Inadequate reporting reduces reproducibility and hampers meaningful cross-study comparison. Greater compliance with TRIPOD-AI and CLAIM frameworks [16,43] is essential to mitigate optimism bias and promote transparent translation into clinical practice.

Importantly, the discordance observed between QUADAS-2 ratings and AI-specific methodological checklist results further underscores the limitations of applying conventional diagnostic accuracy tools alone to AI research. While QUADAS-2 appropriately evaluates bias in patient selection, index testing, and reference standards, it does not capture AI-specific methodological risks such as data leakage, absence of true external validation, lack of calibration analysis, or limited interpretability assessment. Our three-layer assessment framework therefore provides a more nuanced and AI-appropriate appraisal of methodological rigor in diagnostic modeling.

Despite substantial methodological heterogeneity—including variation in imaging protocols, segmentation approaches, feature extraction pipelines, algorithm types, and disease prevalence—statistical heterogeneity was only moderate for sensitivity (I^2^ = 41.8%) and low for specificity (I^2^ = 23.4%), with no evidence of a threshold effect (ρ = −0.117, *p* = 0.776). This suggests that performance variability may reflect dataset composition and validation strategy differences rather than the fundamental instability of AI modeling approaches in OTSCC.

Deeks’ funnel plot asymmetry testing did not demonstrate significant publication bias (*p* = 0.596). However, given the relatively small number of included studies (k = 9), the statistical power of this test to detect subtle small-study effects remains limited.

Despite increasing interest in AI applications for head and neck imaging, research specifically addressing preoperative prediction of occult lymph node metastasis (OLNM) and DOI in OTSCC remains at an early stage. Only twelve eligible studies were identified, and nine provided sufficient data for bivariate meta-analysis. Key limitations include limited external validation, inconsistent calibration reporting, heterogeneity in outcome definitions (OLNM vs. general LNM), small test-set sizes, and exclusive reliance on retrospective designs. Furthermore, 2 × 2 contingency tables were back-calculated in most studies, potentially introducing rounding-related imprecision. Individual patient data were unavailable, precluding patient-level threshold optimization or subgroup analysis by tumor thickness or grade.

To enhance clinical applicability, future research should prioritize: (i) prospective multicenter external validation across heterogeneous scanners and populations; (ii) standardized reporting of calibration metrics and decision-curve analysis; (iii) direct comparison with expert radiologist interpretation to demonstrate incremental value; (iv) robust multimodal modeling strategies; (v) integration of interpretable AI techniques; and (vi) strict adherence to the TRIPOD-AI and CLAIM standards [16,43]. Such measures are necessary to ensure the responsible and methodologically sound translation of AI-assisted diagnostic tools into routine dental and oral oncology practice.

## Figures and Tables

**Table 2:** OLNM definition harmonization and subgroup classification (N = 12).

Study	Endpoint Definition	Clinical Population	Subgroup	Classification Rationale
Yuan et al. [30]	Lymph node metastasis defined as pN+ on elective neck dissection histopathology in clinically node-negative (cN0) patients.	cN0, early-stage (cT1–T2)	OLNM	cN0-restricted population with immediate postoperative histopathological confirmation of occult lymph node metastasis; classified as OLNM.
Kubo et al. [24]	Cervical lymph node metastasis defined as pathologically confirmed nodal involvement detected either at elective neck dissection or within 1-year follow-up in cN0 patients.	cN0, early-stage	OLNM	cN0-restricted cohort with delayed detection of cervical nodal metastasis within a predefined follow-up period; considered conceptually equivalent to OLNM and included in primary analysis, with additional sensitivity evaluation.
Han et al. [23]	Lymph node metastasis defined as pN+ on surgical histopathology in a cN0-restricted cohort.	cT1–T2, multi-center pooled	OLNM	cN0-restricted population with immediate histopathological confirmation; validation strategy classified as internal, as training and validation data were derived from a pooled dataset prior to splitting.
Lan et al. [25]	Lymph node metastasis defined as histopathologically confirmed nodal involvement in clinically node-negative patients.	cN0, early-stage; multi-center	OLNM	cN0-restricted, multicenter cohort; the only study employing true external validation using an independent institutional dataset with different MRI scanners.
Kudoh et al. [17]	Cervical lymph node metastasis defined as surgically confirmed or clinically detected nodal involvement in a mixed cN0/cN+ population.	Mixed cN0/cN+	LNM	Mixed cN0/cN+ population including surgically confirmed and late-detected metastases; classified as general LNM according to prespecified decision rules.
Zhong et al. [31]	Lymph node metastasis defined as pN+ on surgical histopathology in a mixed-stage cohort.	Mixed cN0/cN+	LNM	Mixed cN0/cN+ cohort; although cN0-specific results were reported, independently extractable 2 × 2 data were unavailable; full-cohort data were therefore used for quantitative synthesis.
Vidiri et al. [27]	Primary endpoint: lymph node metastasis defined as pN+ on surgical histopathology; secondary endpoint: DOI/pT stage prediction.	Mixed cN0/cN+	LNM	Mixed cN0/cN+ population; LNM prediction model included in primary analysis; DOI/pT model summarized separately in narrative form.
Liu et al. [26]	Lymph node metastasis defined as histopathologically confirmed nodal involvement in a mixed T/N stage cohort	Mixed stages (all T/N), multi-sequence MRI	LNM	Mixed-stage cohort including all T and N stages; cN0-specific 2 × 2 data were not independently extractable; classified as general LNM per decision rule #2.
Wang et al. [28]	Lymph node metastasis defined as pathologically confirmed nodal involvement; clinical nodal status incorporated as a model input variable.	Mixed cN0/cN+	LNM	Mixed cN0/cN+ population; predictive model incorporated clinical lymph node status as a covariate; classified as general LNM.
Committeri et al. [21] ^†^	Lymph node metastasis prediction as reported; independently extractable 2 × 2 contingency data not provided.	Mixed	—	Excluded from the primary meta-analysis due to high risk of bias (Index Test and Flow & Timing domains of QUADAS-2), absence of an independent validation set, and lack of directly reported 2 × 2 contingency data.
Csüry et al. [22] ^†^	Lymph node metastasis predicted using postoperative histopathologic whole-slide imaging features rather than preoperative imaging data.	Mixed	—	Excluded from the primary quantitative synthesis because model input was based on postoperative histopathology rather than preoperative imaging; included in extended sensitivity analysis (k = 11).
Wang et al. [29] ^†^	Lymph node metastasis prediction as reported; insufficient independently extractable diagnostic contingency data for quantitative synthesis.	Mixed	—	Included in systematic review only; insufficient independently extractable 2 × 2 data for primary quantitative synthesis.

**Decision rules:** (1) Studies restricted to clinically node-negative (cN0) populations with histopathologically confirmed lymph node metastasis were classified as OLNM. (2) Studies including mixed cN0/cN+ populations were classified as general LNM. (3) When both overall and cN0-specific results were reported, independently extractable cN0-specific data were prioritized for quantitative synthesis. (4) Studies with DOI or pT staging as the primary outcome were summarized narratively and excluded from the primary quantitative synthesis. Studies marked with ^†^ indicate studies excluded from the primary meta-analysis. SA, sensitivity analysis; SR, systematic review.

**Table 3:** QUADAS-2 risk of bias assessment for individual studies (N = 12).

Study	Patient Selection	Reference Standard	Index Test	Flow & Timing
Yuan et al. [30]	Low	Low	Low	Low
Kubo et al. [24]	Low	Low	Unclear	Low
Zhong et al. [31]	Low	Low	Low	Low
Kudoh et al. [17]	Low	Low	Unclear	Low
Vidiri et al. [27]	Low	Low	Low	Unclear
Han et al. [23]	Low	Low	Low	Low
Liu et al. [26]	Low	Low	Low	Low
Wang et al. [28]	Low	Low	Low	Low
Lan et al. [25]	Low	Low	Low	Low
Committeri et al. [21] ^†^	Low	Low	High	High
Csüry et al. [22] ^†^	Low	Low	Unclear	Unclear
Wang et al. [29] ^†^	Unclear	Low	Unclear	Low
**Low risk, *n*/12 (%)**	**11 (91.7%)**	**12 (100%)**	**7 (58.3%)**	**9 (75.0%)**

**Risk of bias ratings**: **Low** = low risk; **Unclear** = insufficient information; **High** = high risk of bias. Studies marked with ^†^ indicate studies excluded from the primary meta-analysis (k = 9). All studies had low applicability concerns across all domains. **Reasons for ratings other than low risk**: Patient Selection—Wang et al. [29]: insufficient reporting of consecutive enrollment. Index Test—Kubo et al. [24]: SMOTE applied prior to cross-validation split; Kudoh et al. [17]: threshold determination unclear; Committeri et al. [21]: no independent test set; Csüry et al. [22]: histopathology methodology unclear; Wang et al. [29]: validation strategy unclear. Flow & Timing—Vidiri et al. [27]: incomplete follow-up description; Committeri et al. [21]: no independent verification; Csüry et al. [22]: timing of assessment unclear.

**Table 4:** Characteristics and diagnostic performance of the included AI models for lymph node metastasis prediction in OTSCC.

Study	N	Age	Imaging	Outcome	Best Model	SE	SP	AUC	Validation	Subgroup	Analysis
Yuan et al. [30]	68	NR	MRI	OLNM	ANN	0.633	0.821	0.800	5-fold CV	OLNM	Primary (k = 9)
Kubo et al. [24]	62	NR	CT	OLNM	SVM	0.696	0.713	0.720	LOOCV	OLNM	Primary (k = 9)
Zhong et al. [31]	161	NR	CT	LNM	RF	0.931	0.765	0.850	10-fold CV	LNM	Primary (k = 9)
Kudoh et al. [17]	40	66 ± 14	PET/CT	LNM	Thresholding	0.650	0.700	0.790	5-fold CV	LNM	Primary (k = 9)
Vidiri et al. [27]	36	61	MRI	LNM	RF	0.600	0.714	NR	Random split	LNM	Primary (k = 9)
Han et al. [23]	125	NR	CECT	OLNM	LightGBM	0.750	0.941	0.824	Random split	OLNM	Primary (k = 9)
Liu et al. [26]	25 ^†^	56.9 ± 11.3	MRI	LNM	SVM	0.625	0.824	0.868	Train/test	LNM	Primary (k = 9)
Wang et al. [28]	80	NR	MRI	LNM	MLP	0.529	0.870	0.747	Random split	LNM	Primary (k = 9)
Lan et al. [25]	319	NR	MRI	OLNM	ResNet50 + Rad	0.821	1.00	0.878	External	OLNM	Primary (k = 9)
Committeri et al. [21] ^†^	≈53	NR	Mixed	LNM	CIDT	NR	NR	NR	None (resubst.)	—	SA only (k = 11)
Csüry et al. [22] ^†^	211	NR	Histopath	LNM	RF/SVM	NR	NR	0.830	5-fold CV	—	SA only (k = 11)
Wang et al. [29] ^†^	NR	NR	Mixed	LNM	Logistic	NR	NR	NR	NR	—	SR only

**Abbreviations:** ANN, artificial neural network; CECT, contrast-enhanced CT; CIDT, chi-squared interaction decision tree; CLNM, cervical lymph node metastasis; CV, cross-validation; LNM, lymph node metastasis; LOOCV, leave-one-out CV; MLP, multilayer perceptron; NR, not reported; OCLNM, occult cervical LNM (Kubo et al. [24]; equivalent to OLNM); OLNM, occult LNM; Rad, radiomics; RF, random forest; SA, sensitivity analysis; SR, systematic review; SVM, support vector machine. ^†^ Test set only (total N = 400). SE and SP values represent back-calculated estimates from 2 × 2 contingency tables used in the meta-analysis; these may differ from values reported in original publications. Studies marked with ^†^ were included in the sensitivity analysis or systematic review only and were excluded from the primary meta-analysis (k = 9).

## Data Availability

The data analyzed in this study were derived from previously published studies, which are cited within the article. Extracted data and methodological details are provided in the article and its Supplementary Materials. Further inquiries can be directed to the corresponding author.

## Supplementary Materials

The following supporting information can be downloaded at: https://www.mdpi.com/article/10.3390/diagnostics16050774/s1.

## Appendix A

**Table A1:** Complete Electronic Search Strategy for PubMed. The electronic search strategy was structured into three predefined concept blocks: (1) artificial intelligence and machine learning; (2) oral tongue and oral cavity malignancies; and (3) predictive outcomes related to lymph node metastasis and depth of invasion. Within each block, Medical Subject Headings (MeSH) terms and free-text keywords were combined using the Boolean operator OR. The three blocks were then combined using AND. No date or language restrictions were applied. The search was limited to human studies. The final search was conducted on 15 January 2026.

PubMed (MEDLINE)		
Step	Search Query	Results
#1 AI/ML	(“artificial intelligence”[MeSH Terms] OR “machine learning”[MeSH Terms] OR “deep learning”[MeSH Terms] OR “neural networks, computer”[MeSH Terms] OR “artificial intelligence”[Title/Abstract] OR “machine learning”[Title/Abstract] OR “deep learning”[Title/Abstract] OR “radiomics”[Title/Abstract] OR “radiomic”[Title/Abstract] OR “texture analysis”[Title/Abstract] OR “convolutional neural network*”[Title/Abstract] OR “random forest”[Title/Abstract] OR “support vector machine”[Title/Abstract] OR “transfer learning”[Title/Abstract])	—
#2 Disease	(“tongue neoplasms”[MeSH Terms] OR “mouth neoplasms”[MeSH Terms] OR “oral tongue”[Title/Abstract] OR “tongue squamous cell carcinoma”[Title/Abstract] OR “OTSCC”[Title/Abstract] OR “oral squamous cell carcinoma”[Title/Abstract] OR “OSCC”[Title/Abstract] OR “oral cavity cancer”[Title/Abstract] OR “tongue cancer”[Title/Abstract] OR “oral cavity neoplasm*”[Title/Abstract] OR “lingual squamous cell carcinoma”[Title/Abstract])	—
#3 Outcomes	(“lymphatic metastasis”[MeSH Terms] OR “neoplasm staging”[MeSH Terms] OR “lymph node metastasis”[Title/Abstract] OR “lymph node metastases”[Title/Abstract] OR “occult metastasis”[Title/Abstract] OR “occult metastases”[Title/Abstract] OR “cervical lymph node”[Title/Abstract] OR “cervical nodal”[Title/Abstract] OR “depth of invasion”[Title/Abstract] OR “DOI”[Title/Abstract] OR “tumor invasion”[Title/Abstract])	—
#4 Combined	#1 AND #2 AND #3	80
**Summary**		
**Database**	**Records**	**After Dedup**
PubMed (MEDLINE)	80	—
Total	80	80

A total of 80 records were identified through the electronic search and screened by title and abstract. Reference lists of all included articles were manually reviewed to identify additional potentially relevant studies. Grey literature sources (e.g., conference proceedings, preprints, and clinical trial registries) were not systematically searched. Given that AI- and radiomics-based predictive models require detailed methodological reporting—including model architecture, validation design, and performance metrics—such information is frequently incomplete or unavailable in non-peer-reviewed sources. Therefore, only peer-reviewed publications were considered eligible to ensure methodological transparency and reproducibility. The search was limited to human studies. No date or language restrictions were applied.

**Table A2:** Integrated methodological quality assessment combining QUADAS-2 and AI-specific checklist items.

Study	QUADAS-2 Domains					AI-Specific Items								Discordance
	PS	Ref Std	Idx Test	F&T	All Low?	ICC	Imbal	ExtVal	Calib	DCA	Clin Cmp	Rpt Gdln	Code	
Yuan et al. [30]	Low	Low	Low	Low	Yes	Yes	No	No	No	No	No	No	No	All QUADAS-2 domains low risk; 7/8 AI criteria unmet.
Kubo et al. [24]	Low	Low	Unclear	Low	No	No	Yes	No	No	No	No	No	No	
Zhong et al. [31]	Low	Low	Low	Low	Yes	Yes	No	No	No	No	Yes	No	Yes	All QUADAS-2 domains low risk; 5/8 AI criteria unmet.
Kudoh et al. [17]	Low	Low	Unclear	Low	No	No	No	No	No	No	No	No	No	
Vidiri et al. [27]	Low	Low	Low	Unclear	No	Yes	Yes	No	No	No	No	Yes	No	
Han et al. [23]	Low	Low	Low	Low	Yes	No	No	No	No	No	No	No	No	All QUADAS-2 domains low risk; 8/8 AI criteria unmet.
Liu et al. [26]	Low	Low	Low	Low	Yes	Yes	No	No	Yes	No	No	No	No	All QUADAS-2 domains low risk; 6/8 AI criteria unmet.
Wang et al. [28]	Low	Low	Low	Low	Yes	No	No	No	Yes	Yes	No	No	No	All QUADAS-2 domains low risk; 6/8 AI criteria unmet.
Lan et al. [25]	Low	Low	Low	Low	Yes	No	Yes	Yes	Yes	Yes	No	Yes	No	Highest AI adherence: 5/8 items fulfilled.
Committeri et al. [21]	Low	Low	High	High	No	No	No	No	No	No	No	No	Yes	QUADAS-2 concerns consistent with AI-specific methodological gaps.
Csüry et al. [22]	Low	Low	Unclear	Unclear	No	No	No	No	No	No	No	No	No	
Wang et al. [29]	Unclear	Low	Unclear	Low	No	Yes	No	No	No	No	No	No	No	

**Abbreviations:** PS, Patient Selection; Ref Std, Reference Standard; Idx Test, Index Test; F&T, Flow and Timing; ICC, intraclass correlation coefficient; Imbal, class imbalance handling; ExtVal, external validation; Calib, calibration assessment; DCA, decision curve analysis; Clin Cmp, clinician comparison; Rpt Gdln, reporting guideline adherence; Code, code and/or data availability. QUADAS-2 domains and AI-specific methodological checklist items are presented in separate column groups. “Yes” indicates fulfillment of the criterion, and “No” indicates absence. The final column summarizes the concordance between QUADAS-2 risk assessment and AI-specific methodological adherence.
